# Severe extensive community acquired aspergillus infection in a full‐term infant accompanied with increases in white blood cell counts and C-reactive protein: a case report

**DOI:** 10.1186/s12887-021-02588-1

**Published:** 2021-03-09

**Authors:** Yanli Wang, Wenjing Chen, Wenshen Wu, Dongling Yu, Huiheng Yan, Xiuzhen Ye

**Affiliations:** 1 Guangdong Women and Children Hospital, No. 13, Guangyuan West Road, Yuexiu District 510000 Guangzhou, China; 2 Dongguan Children’s Hospital, Guangdong Medical University, 523000 Dongguan, China

**Keywords:** Aspergilloma, Full‐term neonate, Community infection, Aspergillus infection, Significant increases in WBC and CRP

## Abstract

**Background:**

Aspergillus infection is more common among premature infants in neonatal intensive care units, who have decreased qualitative immune defenses and need various invasive treatment procedures. It is rare in normal full-term neonates, especially in newborn babies from the community. Moreover, the white blood cell (WBC) count and C-reactive protein (CRP) level may be normal or slightly changed in fungal infections, but the neonate reported in this study had significant increases in WBC and CRP. To the best of our knowledge, this is the first report on a full-term neonate from the community with aspergillus infection accompanied by significant increases in WBC and CRP levels.

**Case presentation:**

A 28-day-old infant, who received empirical antibiotic treatment for 10 days because of neonatal pneumonia, was referred to our neonatal department from the local hospital. The infant had persistent infection and multiple organ failure syndromes. Bronchoscopy and deep sputum smear were performed to identify the pathogen, which confirmed aspergillus infection in the sputum. Fluconazole was immediately administered, but the baby died after three days. Thereafter, an autopsy was performed with parental consent. There were multiple necrotic areas in the lungs and liver, and pathological examination revealed aspergillus.

**Conclusions:**

The present case emphasized that community-sourced aspergillus infection can exist in full-term neonates, with significantly increased WBC count and CRP level. Advanced antibiotics were not effective in this case, and fungal infections should have been considered earlier.

## Background

Aspergillus infection is more common in premature and extremely low birth weight infants in neonatal intensive care units, who have decreased qualitative immune defenses and need various invasive treatment procedures [1, 2]. It is typically rare in normal full-term neonates, especially those from the community. Additionally, the white blood cell (WBC) counts and C-reactive protein (CRP) levels are increased in fungal infections [3].

## Case presentation

A male infant was transferred to our neonatal department from the local hospital on day 26 after birth with persistent infection and multiple organ failure syndromes. The baby was born on October 19, delivered by caesarean section at 39 weeks and two days. The infant’s birth weight was 3.15 kg. He had Apgar scores of 9 and 10 points at 5 and 10 min, respectively, and was vaccinated on day 1 after birth for hepatitis and BCG.

The 25-year-old mother did not have any specific medical history, and did not receive antibiotic treatment or antenatal steroids. The baby was healthy and fed with breast milk and formula after birth.

On day 16 after birth, the infant was taken to a level I hospital because of cough and fever. His body temperature was 38.6℃ and respiratory rate was 62 beats per minute. Antibiotic therapy with penicillin and cefotaxime was initiated due to suspected community-acquired bacterial infection at the local hospital. However, five days later, the baby developed dyspnea with persistent fever, so he was transferred to a level II hospital. On admission, the baby’s body temperature was 38.9℃ and respiratory rate was 72 beats per minute. Hence, nasal continuous positive airway pressure (nCPAP) treatment was given at that hospital, and meropenem and vancomycin were initiated for five days. However, the baby developed progressive dyspnea and low oxygen saturation, for which tracheal intubation and mechanical ventilation were needed. Thereafter, the baby was transferred to our neonatal department with fever and multiple organ failure syndromes.

The clinical examination of the infant on admission at our hospital showed the following: body temperature was 38.3℃, with intubation ,heart rate was 181 beats/min, body weight was 3.8 kg, blood pressure was 95/67 mmHg, pulse oxygen saturation (SpO2) was 86 % with 100 % FiO2.

He was presented with poor response, endotracheal intubation, flat and soft anterior fontanelle with 1.5 cm * 1.5 cm in size, and the bilateral pupils were circular and equal in size, reflecting light. The neck was soft with no resistance. The breath sounds of both lungs were symmetrical, but moist rales were detected in both lungs. The abdomen was flat and soft, with enlarged liver (40 mm below the ribs), not palpable under the spleen rib, with normal bowel sounds. The muscle tension of the limbs was increased. Hugging, holding, foraging and sucking reflexes were not elicited.

Blood test results at the local hospital were as follows: WBC count was 28–45 × 10^9^/L (normal range, < 20 × 10^9^/L), C-reactive protein was 188.2 mg/L (normal range, < 10.0 mg/L), and procalcitonin (PCT) was 2.1 mg/L (normal range, < 0.1 mg/L). The remaining blood test markers were within normal limits: neutrophil granulocytes was 65 % (normal 50–70 %), platelet count (PLT) was 72 × 10^9^/L (normal 100–350 × 10^9^/L). The blood culture was negative.

Blood test results on admission at our hospital were as follows: WBC count was 44 × 10^9^/L and CRP was 548.98 mg/L, which were higher than before, but PLT was lower at 32 × 10^9^/L, PCT was 4.8 mg/L, β-D glucan level was 210 ng/L (normal range, < 11.0). The remaining blood test markers were normal: neutrophil granulocytes was 61 %, IgA was 0.17 g/L, IgG was 2.2 g/L, and IgM was 0.27 g/L. Lymphocyte subset analysis showed: T% = 61.16 %, B% = 6.5 %, NK% = 12.03 %, CD4 + T = 33.33 %, CD8 + T = 29.33 %.

The X-ray results showed that the neonate presented with pneumonia, and with massive consolidation of right lung and atelectasis. Liver color ultrasound showed hyperechoic imaging, and ECG showed sinus tachycardia.

Bronchoscopy and deep sputum smear were immediately performed. The results revealed obvious endobronchitis, necrotic detritus, and pseudo-membrane in the trachea, right main bronchi and branches. The sputum was positive for Aspergillus fumigatus under microscopy. The subsequent blood culture also revealed the presence of Aspergillus fumigatus. Hence, we switched the antibiotics treatment with fluconazole, based on the results of drug sensitivity test. However, the baby died after three days due to clinical deterioration and multiple organ failure.

**Fig. 1:**
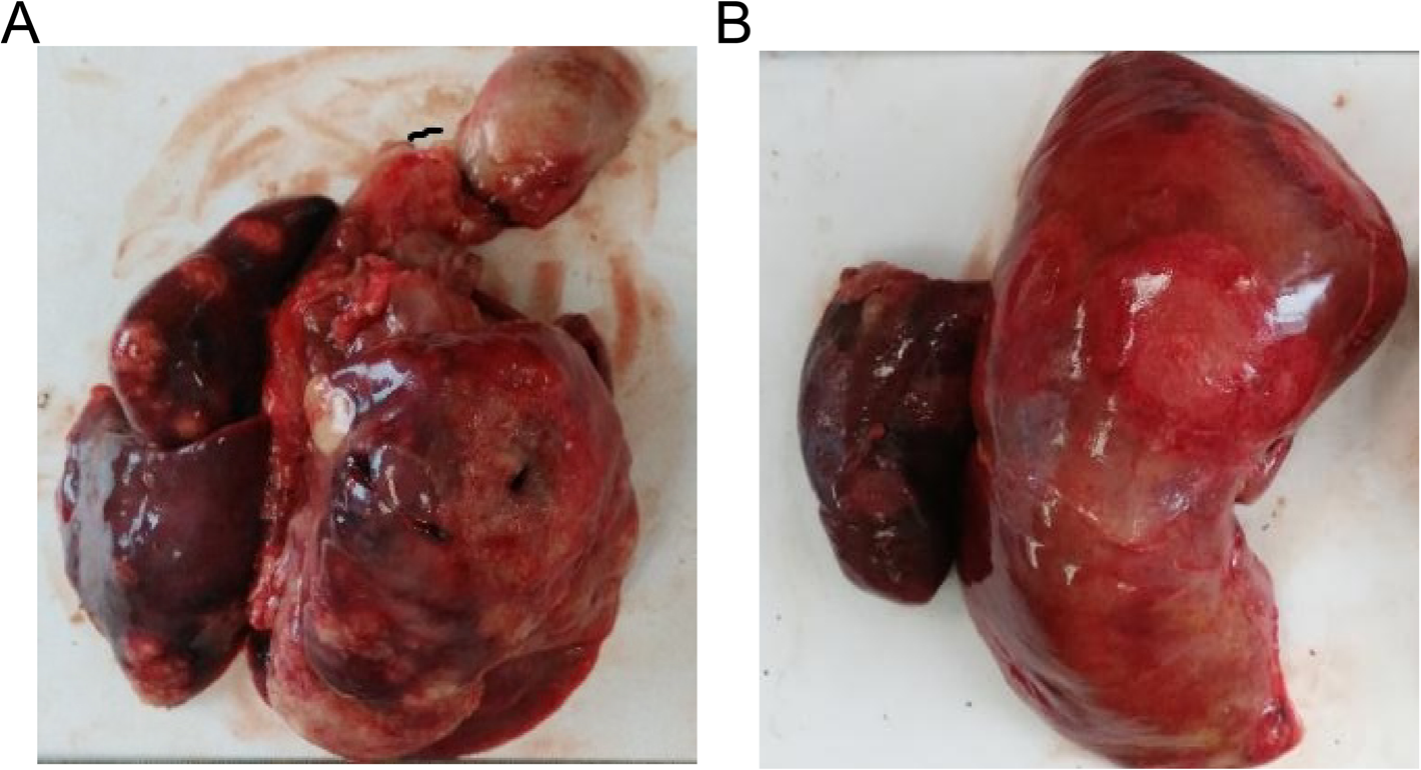
Specimen of liver. **a **Gross specimen of the lung showing multiple friable grey–white necrotic areas. **b** Gross examination of the liver - Multiple nodular and cavitary lesions on the external surface

With the consent of his family, a biopsy was conducted on the infant to ascertain the cause of death. On gross examination, the lungs weighed 280 g, the left lung and right lung measured 90 × 70 × 30 mm and 11 × 8 × 5 mm, respectively. The cut surface of both lungs showed multiple areas with grey–white necrosis (Fig. 1A). The liver weighed 308 g and measured 170 × 80 × 50 mm. The cut surface of the liver showed multiple areas with grey–yellow necrosis (Fig. 1B). The brain weighed 520 g and the cut surface showed grey–yellow necrotic areas at the top of the brain, measuring 15 mm×15 mm. The skin also showed necrotic areas. The heart, kidneys and spleen were grossly unremarkable.

**Fig. 2:**
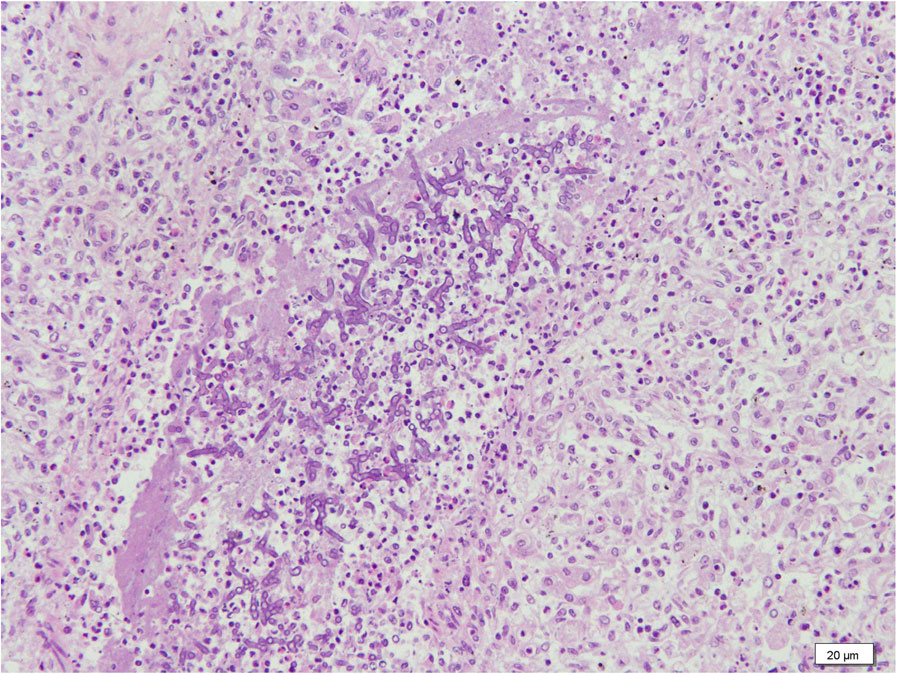
Photomicrophotographs of the lung, which reveal the destruction of intermingle with necrotic debris and fungal hyphae (H&E, 200X)

**Fig. 3:**
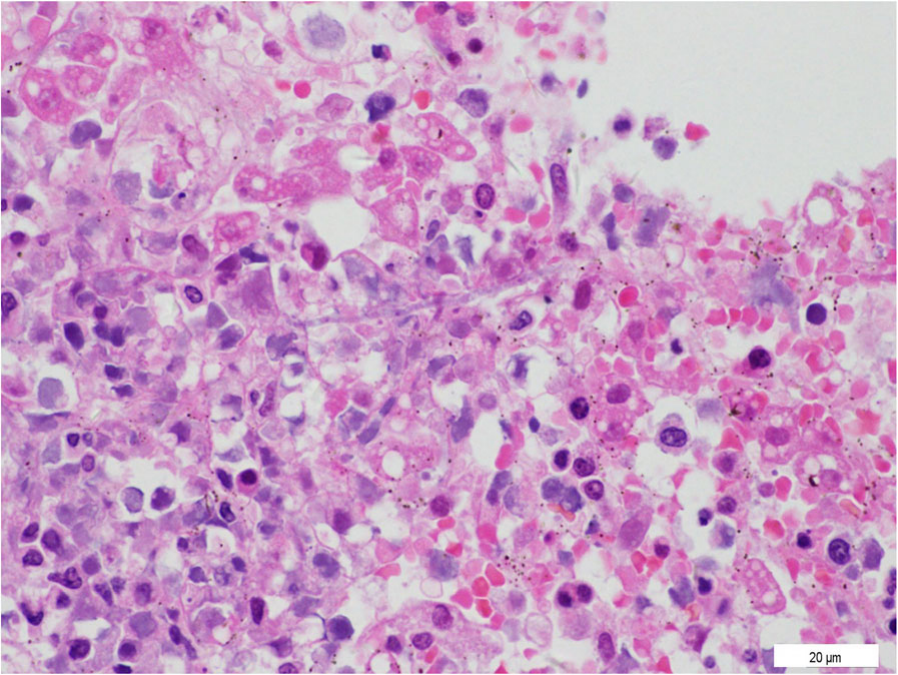
Photomicrophotographs of the liver, which reveal hemorrhage and necrosis with hyphae (H&E, 400X)

**Fig. 4:**
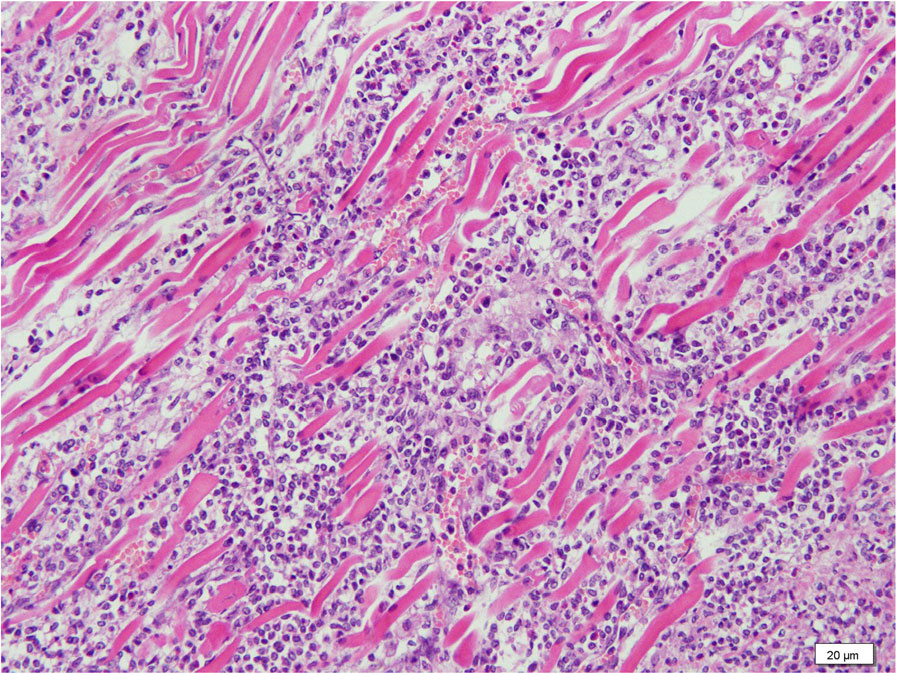
The fungal hyphae were septate with striated muscles, lymphocytes and eosinophils can be seen (H&E, 200X)

**Fig. 5:**
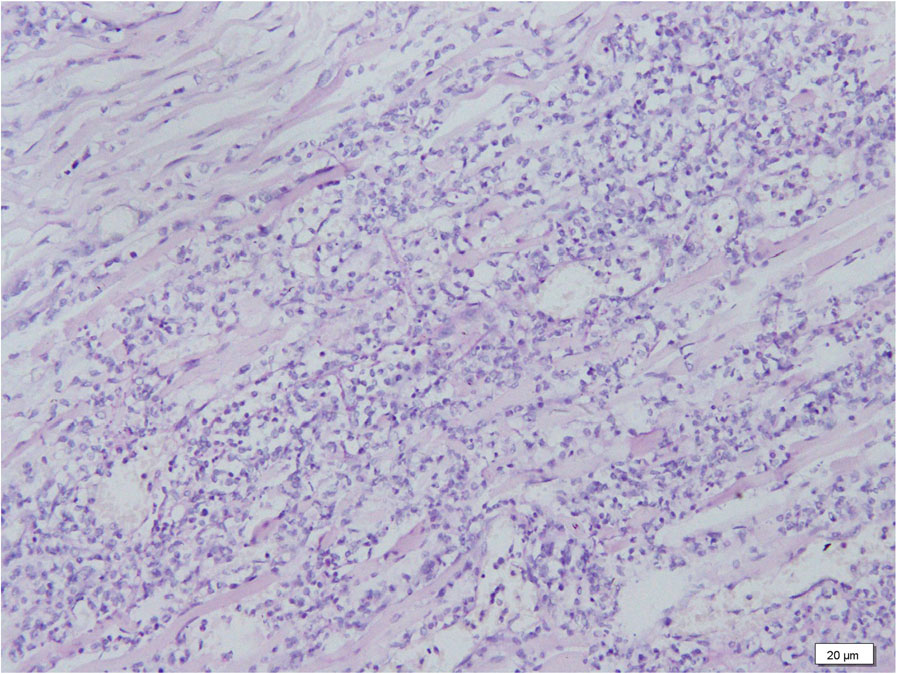
PAS positive (arrows) in striated muscle revealing the presence of fungal hyphae (PAS, 200X)

Then the Olympus Camera (DP21) under the Olympus microscope BX51 with the software of camera was used to investigate pathology. Representative micro-sections from lungs, liver, brain and skin showed extensive multifocal areas of necrosis with acute and chronic inflammatory infiltrates (Figs. 2, 3 and 4), and the presence of fungal hyphae with a morphology similar to Aspergillus fumigatus. These fungal hyphae were also seen with Periodic acid-Schiff (PAS) staining (Fig. 5). Micro-sections examined from the heart, kidneys and spleen were unremarkable. The blood culture was positive for Aspergillus fumigatus. Thus, disseminated invasive aspergillosis was diagnosed.

### Epidemiological investigation

After diagnosis of invasive aspergillosis, we performed an epidemiological investigation to identify the source of Aspergillus fumigatus. Detailed history recording revealed that the baby’s 73-year-old grandmother who lived with the family had a long-term chronic cough. The family lives on the first floor, the room is damp and has no sunshine. The grandmother could not see sunshine during confinement since the doors and windows were closed for a long time. The baby was nursed, cleaned and fed by the sick and coughing grandmother. The family habitually eats pickles and leftovers. Our request to test the grandmother was refused by the family. The family also refused tests for their immune system function. So whether immunological deficiency existed in the baby and the cause of aspergillosis remained unclear.

## Discussion and conclusions

Neonatal invasive fungal disease is mainly caused by Candida spp [4]. Aspergillosis is often localized to the skin and soft tissues [5]. Primary cutaneous aspergillosis (PCA) is common in premature infants because of defects in the skin barrier [6]. Neonatal aspergillosis is one of the most common causes of morbidity and mortality in premature neonates [7]. The clinical signs and symptoms of aspergillosis infection are often nonspecific. The patient in our report was a full-term baby from the community, without any risk factors. He had invasive Aspergillus fumigatus infection, which was an unusual case.

The major risk factor for invasive aspergillosis is the quantitative or qualitative deficiency of neutrophil granulocytes. Persistent febrile neutropenia is a high-risk factor in the early phase, but the neutrophil granulocytes were normal in this case. Initially, the infant presented with significantly increased WBC count and CRP level, which indicated bacterial infection or sepsis, and neutrophils and PCT were slightly increased. These findings led to confusion and misdiagnosis. However, some studies have reported Aspergillus fumigatus infection cases accompanied by high WBC and CRP along with low PCT [8–10]. So whether fungal infection should be considered if a baby has high WBC count and CRP level needs further study.

In summary, fungal infection should be considered if an empirical antibiotic treatment is not effective in an infant with high WBC and CRP, and low PCT. Also, more cultures are required from different samples or other means to achieve an early diagnosis.

## Data Availability

The datasets used and analyzed during the current study are available from the corresponding author on reasonable request.
